# Evaluation of Efficacy of Various Surface Conditioning Methods on the Repair Bond Strength of Composite to Different Fracture Types of Zirconia Ceramics

**DOI:** 10.1155/2021/5537761

**Published:** 2021-05-21

**Authors:** Serdar Polat, Emre Tokar, Neset Volkan Asar, Omer Kirmali

**Affiliations:** ^1^Gazi University, Faculty of Dentistry, Department of Prosthodontics, Ankara, Turkey; ^2^Akdeniz University, Faculty of Dentistry, Department of Prosthodontics, Antalya, Turkey

## Abstract

The aim of this study was to investigate the effects of different surface treatment methods on shear bond strength between composite resin and different levels of zirconia ceramic. Laser surface-conditioning procedures have been reported as effective method to increase repair bond strength of composite to zirconia ceramics. Detailed information of effects of Er,Cr:YSGG laser treatment with different pulse rates on the zirconia ceramics is lacking in the literature. 120 disc-shaped specimens were prepared including zirconia, veneering ceramic, and 50% veneering ceramic-50% zirconia surfaces. Four different surface treatments were applied to the specimens. These were grinding with diamond bur, sandblasting, and short and long pulse rates of Er,Cr:YSGG laser irradiation. An intraoral ceramic repair kit was used to repair specimens, and shear bond strength was performed on the composite resin to each specimen. The highest mean bond strength was seen in the veneering ceramic surface that was ground using a diamond bur, and the lowest mean bond strength value was observed in the same surface that was treated with long pulse laser irradiation. The sandblasting with alumina particles exhibited lower mean repairing bond strength among the rest of used methods in this study for the group which contained half of the veneering ceramic and half of the zirconia. Sandblasting and Er,Cr:YSGG laser using surface treatment procedures obtained appropriate bond strength for the group that included 50% veneering ceramic-50% zirconia, because of no significant differences observed among the applied surface conditioning methods in this group.

## 1. Introduction

Outstanding esthetics and biocompatibility features of all-ceramic restorations are enabled to find place widely used in restorative dentistry. All-ceramic crowns, for example, aluminum-based porcelains, zirconium oxide ceramics, and leucite or lithium-disilicate-reinforced glass ceramics, can be considered an alternative treatment option to porcelain fused to metal fixed partial dentures in daily clinical practice of restorative dentistry [1, 2]. Among these materials, zirconia has superior mechanical properties compared to conventional ceramics, because that is the most stable and high-strength ceramic material. It has 900 MPa flexural strength and 9 MPa/m^1/2^ fracture toughness values [1].

Zirconia core ceramics have been demonstrated to be used more safely as framework from single crowns to multiunit bridgeworks. The zirconia core materials are veneered with a stratified feldspathic porcelain to achieve superior esthetics. Although high survival rates of the zirconia ceramics (73.9%-100% after 2-5 years) have been reported in anterior and posterior fixed partial dentures [3–5], clinicians still encounter some technical complications such as framework fracture and porcelain chipping or extended crack of the porcelain.

Fracture in the zirconia framework has been reported as a rarely observed complication with an annual failure rate ranging from 1.88% to 4.24%. On the other hand, minor chipping and complete fracture of the porcelain have been most frequent technical complication. Chipping or crack of porcelain layer can be fracture originating from either zirconia core-porcelain junction or the porcelain itself. Porcelain cracks have been referred to tensile stresses that result from flaws regardless of internal or external characteristics. Stresses in the veneering porcelain throughout the cooling procedure and incompatibility of the coefficient of thermal expansion between zirconia framework and veneering porcelain are other possible causes for beginning and propagation of the crack [6].

When a fracture occurs in veneer ceramics, intraoral repair methods are easier than complete replacement or application of extraoral repair techniques. Also, that is less traumatic for the patient and do not require additional time, efforts, and costs [7, 8].

Intraoral porcelain repair kits include a bonding system to bond dental composite resin to a fractured porcelain restoration [9]. Strong and effective bonding between resin and porcelain depends on the chemical bond in this interface, and surface-roughening procedures on ceramic material enhance the micromechanical retention [10]. In that case, various treatments include sandblasting with Al_2_O_3_ particles, acid etching, liner application, grinding with diamond disc, and laser application. Among these methods, benefits of laser application, namely, erbium:yttrium aluminum garnet (Er:YAG), carbon dioxide (CO_2_), neodymium-doped yttrium garnet (ND:YAG), and erbium chromium:yttrium scandium gallium garnet (Er,Cr:YSGG) lasers, as a chair-side application have become popular and commonly used method recently [7, 8, 11–25].

This study is aimed at investigating the efficacy of Er:Cr:YSGG laser application with short and long pulse durations in comparison to other surface roughening procedures for the repair of zirconia ceramics with different surface configurations. The null hypothesis of this study was that the laser irradiation and sandblasting applications have no effect on the repair bond strength of the different level of zirconia ceramics.

## 2. Materials and Methods

Totally, 120 disc-shaped specimens (5 mm in diameter and 2 mm in height) were manufactured to analyze the shear bond strength of porcelain repair system to various ceramic surfaces treated with different methods. 3 main groups were determined, so that 40 discs were randomly selected for each group. The following surface configurations were defined for these groups: *Group I*: testing surfaces of the specimens were merely fabricated from zirconia (zirconia 100%) (ICE Zirkon Translucent, Zirkonzahn GmbH, Gais, Germany). (Zirconia Group); *Group II*: testing surfaces of the specimens were only fabricated from veneering ceramic (veneering ceramic 100%) (Vita VMK Master, Vita Zahnfabrik H. Rauter GmbH & Co. KG, Bad Säckingen, Germany). (Veneering Group); *Group III*: testing surfaces of the specimens were fabricated from 50% veneering ceramic and 50% zirconia (Zirconia-Veneering Group).

### 2.1. Preparation of the Specimens

**Figure 1:**
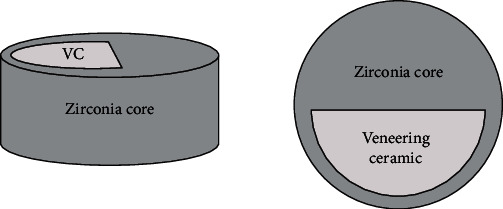
Illustration of 50% zirconia-50% veneering ceramic specimen.

Disc-shaped zirconia specimens were milled using a machine-milling technique from the presintered zirconia green blocks. Afterwards, the final sintering was performed at 1500°C according to manufacturer's recommendations. 40 specimens were left as only zirconia specimens (Group I). For fabricating 40 zirconia specimens coated with veneering ceramics (Group II), the veneer ceramic powder and liquid were mixed, and the slurry obtained was applied into the disc shaped metal mold that wrapped around the preprepared zirconia discs. This metal mold had sufficient space in it to provide the veneering ceramic as thick as the zirconia specimens that was coated. Then, zirconia and veneering ceramic assembly was fired in a computer-assisted oven owing to the instructions manual. Subsequently, a slot measuring 1 mm in depth was prepared into half of the testing surface of the 40 remaining zirconia specimens as was shown in Figure 1. After mixing veneer ceramic powder with liquid, the slurry was condensed, dried, and fired following procedures recommended by the manufacturers. Thus, 40 specimens having surfaces 50% zirconia and 50% veneering ceramics (Group III) were obtained (Figure 1). All specimens were embedded into cylindrical molds that were filled with autopolymerizing acrylic.

40 specimens of each group were randomly distributed into 4 subgroups of 10 discs each. Different surface treatments were applied to the testing surfaces of specimens of each subgroup. Surface treatments applied were as follows: *Subgroup I*: control group, surface grinding with wheel grinding diamond bur (Swisstec WH40-1.5, Diatech, Charleston, SC, USA) (10 strokes); *Subgroup II*: airborne particle abrasion group, sandblasting with Rondo flex plus 360 (intraoral sandblasting device). 50 *μ*m Al_2_O_3_ particles were applied at an air pressure of 4 bar and water pressure of 1.5 bar for 20 s at a distance of 1 mm, perpendicularly to the surface; *Subgroup III*: laser application with long pulse, bonding surfaces of the specimens were irradiated by the Er,Cr:YSGG laser (Millennium; Biolase Technology, Inc., San Clemente, CA) at a 2.78 *μ*m wavelength. The optical fiber of laser (600 *μ*m in diameter, 6 mm in length) was placed perpendicularly to the surface at a distance of 10 mm. The pulse duration was 200 *μ*s with a repetition rate of 20 Hz (pulses/s), and the pulsed laser-powered hydrokinetics, energy, and power densities were 5.6 J/cm^2^ and 111 W/cm^2^ at 1.5 W, respectively. Water and air flows of 50% and 50%, respectively, were used continuously during the 20 s irradiation; and *Subgroup IV*: laser application with short pulse, bonding surfaces of the specimens were irradiated by Er,Cr:YSGG laser (Millennium; Biolase Technology, Inc., San Clemente, CA) with a 2.78 *μ*m. The pulse duration was 140 *μ*s with a repetition rate of 20 Hz (pulses/s), while the other conditions of this laser were similar to that of Subgroup III.

**Figure 2:**
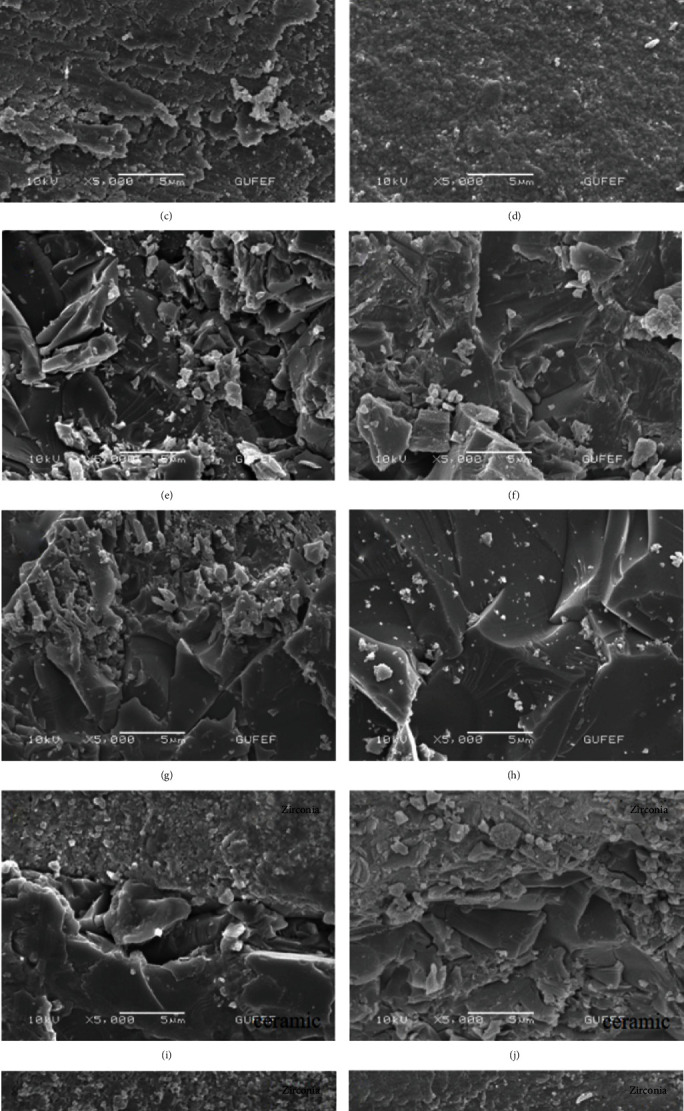
SEM images of the specimens after the surface treatments at a magnification of 5000x. (a) Zirconia-control (Group I-Subgroup I). (b) Zirconia-sandblasting (Group I-Subgroup II). (c) Zirconia-long-pulse laser (Group I-Subgroup III). (d) Zirconia-short-pulse laser (Group I-Subgroup IV). (e) Ceramic-control (Group II-Subgroup I). (f) Ceramic-sandblasting (Group II-Subgroup II). (g) Ceramic-long-pulse laser (Group II-Subgroup III). (h) Ceramic-short pulse laser (Group II-Subgroup IV). (i) Zirconia/ceramic-control (Group III-Subgroup I). (j) Zirconia/ceramic-sandblasting (Group III-Subgroup II). (k) Zirconia/ceramic-long-pulse laser (Group III-Subgroup III). (l) Zirconia/ceramic-short-pulse laser (Group III-Subgroup IV).

After the surface treatments were completed, the surfaces were examined with the scanning electron microscope (JEOL, JSM, 6060) in order to observe the topographic surface changes. The samples were first dried, and a sputter-coating device was used to sputter-coat with gold and palladium. The SEM images were then obtained using an electron microscope with a magnification of 1000x, 2000x, and 5000x. SEM pictures with 5000x magnification are shown in Figure 2.

After the SEM examination, all specimens were bathed in ultrasonic cleaner with 96% isopropyl alcohol (Sigma-Aldrich, St. Louis, MO) for 3 mins. Then, the intraoral ceramic repair kit (Clearfil Repair, Noritake, Kuraray, Japan) and composite material (Clearfil Majesty Esthetic, Noritake, Kuraray, Japan) were used to apply to surface of the specimens following the manufacturer's recommendations. In short, following the instructions, the conditioned testing surfaces of the specimens involving veneering ceramic (Group II and III) were etched with 40% phosphoric acid (K-etchant Gel; Kuraray Co. Ltd., Osaka, Japan) for 5 s (no need for zirconia surfaces) and rinsed with water for 15 s. Following air drying, the Clearfil SE Bond Primer and Clearfil Porcelain Bond Activator were mixed at a 1 : 1 ratio and put on to the surfaces for 5 s and air thinned. The SE Bond was applied, air dried, and photopolymerized for 10 s. The composite resin material (Clearfil Majesty Esthetic, Noritake, Kuraray, Japan) was applied to the specimen surfaces with the aid of the Teflon mold which had 2x2x2 mm dimension. The mold was incrementally filled up, and each layer was light-polymerized for 20 s at a distance of 1 mm using a light-polymerizing unit (BlueLEX BT-150, Monitex Industrial Company Ltd, New Taipei City, Taiwan) with an output power of 11 W and 1.2 A. Before the shear bond test, specimens were stepped in 37°C distilled water for 24 h.

### 2.2. Shear Bond Strength Test

**Figure 3:**
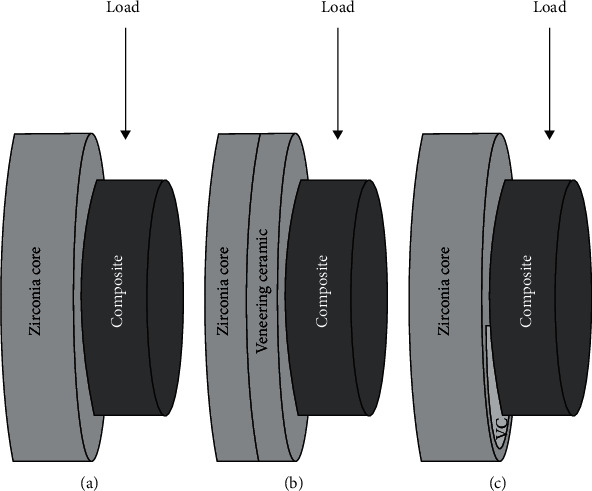
Illustration of shear bond strength test of the specimens. (a) Group I (zirconia). (b) Group II (veneering ceramic). (c) Group III (zirconia and veneering ceramic).

Each specimen was placed in a metal holder in a universal testing machine (Shimadzu AG-X; Tokyo, Japan). Loading parallel to the long axis of the specimen was applied at the interface between composite block and testing surface at a 0.5 mm/min crosshead speed until fracture (Figure 3). The maximum load at failure or delamination of the composite blocks was noted. Shear bond strength was calculated dividing failure load (N) by bonding area (mm^2^).

Following the shear bond test, observation on the fractured surfaces was carefully carried out, and the failure modes in the interfaces were determined by a single researcher. The observed failure modes were adhesive, cohesive failures, and mixed of them. Completely separated composite resin from the zirconia surface is determined as adhesive failure; the complete fracture in the composite resin is defined as cohesive failure, and the failure which includes both of the failures (adhesive and cohesive failures) is rendered as mixed failure.

### 2.3. Statistical Analysis

The Shapiro-Wilk normality analysis and the homogeneity test of variance analysis revealed that group distributions were normal. Through the results of those tests, the parametric one-way analysis of variance (one-way ANOVA) was performed among the groups applying a statistical analysis software program (PSPP 1.0.1, GNU, FSF Inc., Boston, MA, USA). *p* < 0.05 was considered statistically significant.

## 3. Results

The shear bond strength values including means and standard deviations are presented in Table 1. In all groups, surface grinding (Subgroup I) resulted into the highest mean shear bond strength values. For Group I, the lowest value was detected for Subgroup III and statistically significant differences were observed among Subgroups I, II, and III (*p* < 0.05). Differences between the Subgroups II and III and the Subgroups I and IV were not statistically significant (*p* > 0.05). According to results of Group II, the Subgroup IV showed the lowest bond strength value, and there were statistically significant differences between the values of the Subgroups I and III and between the Subgroups I and IV (*p* < 0.05). The Subgroup II illustrated any statistically significant differences among the other subgroups. In Group III, the Subgroup II presented the lowest bond strength value, but this was not statistically significant among all other subgroups (*p* > 0.05).

Moreover, the failure modes are assessed and presented in Table 2. The majority of failure analysis revealed as adhesive failure in the Group I. 80% of failures were adhesive for Subgroups I and II. All failures occurred in Subgroups III and IV were adhesive. In Group II, all failures occurred predominantly in cohesive in the composite resin (90% for Subgroup I, 80% for Subgroup II, and 70% for Subgroups III and IV). In Group III, with respect to Subgroup I, 50% mixed, 40% cohesive, and 10% adhesive failures occurred. In Subgroups II and III, 60% mixed and 40% cohesive failures were observed. In Subgroup IV, 80% mixed and 20% cohesive failures were detected.

Evaluation of the SEM images was performed after the surface treatment procedures (grinding, sandblasting, long-pulse, and short-pulse Er,Cr:YSGG laser irradiation) under at a magnification of 5000x (Figure 2). Diverse roughness patterns were observed at the SEM evaluation of conditioned surfaces. For the Group I (zirconia surface), parallel scratches were shown compatible with direction of the grinding bur for the Subgroup I, irregular morphological pattern was observed at the Subgroup II. Parallel shallow fissures and scratches were seen at the Subgroup III, and homogeneous surface roughness and microretentive grooves were detected for the Subgroup IV. For the Group II (ceramic surface), irregular surface pattern and wide cracks with deep fissures and high ridges were observed at the Subgroup I. Deep fissures and irregular topography were shown at the Subgroup II. Uniform deep fissures and grooves were seen at Subgroup III. Shallow fissures and grooves were illustrated on the surface of Subgroup IV. For the Group III (50% zirconia-50% veneering ceramic), the subgroups demonstrated similar surface patterns to Groups I and II regarding the type of material.

## 4. Discussion

Outstanding mechanical properties such as high fracture toughness and natural appearance are a key to increase clinical application of zirconia ceramics. Despite the popularity and high clinical success rates of zirconia-based fixed restorations, a considerable amount of veneering ceramic material fracture has been reported by some clinical studies [26–28]. The unexpected fracture of veneer ceramic can lead to dissatisfaction and disappointment in both patients and clinicians. The repair of fractured ceramic instead of fabricating the new one, especially applying one of the intraoral repairing methods, may be more acceptable, easy, less traumatic, and more satisfactory, if the repaired site functions properly through a long-time period [7].

The clinical achievement is frequently reliant on the integrity of the bonding strength in the interface of the fractured surface of zirconia/veneering ceramic and repairing material. Thus, several researches have been conducted related to outcomes of Er,Cr:YSGG laser application to zirconia ceramics, due to the aiming improvement of bond strength between the resin composite and the zirconia surface [21–25]. In this manner, the current study is aimed at assessing the effectiveness of various surface treatment methods on the shear bond strength between the repairing material, composite resin, and zirconia and veneering porcelain. The null hypothesis of the current study was accepted. Various pulse rates of Er,Cr: YSGG laser and sandblasting applications did not improve repair bond strength of not only the zirconia but also veneering porcelain compared to control group, solely grinding with diamond bur.

The shear bond test is one of the most commonly used bond strength tests [7–10, 16, 20, 22–24, 29, 30], since shear stresses are believed to be major stresses involved in in vivo bonding failures of restorative materials. Surface roughness and bond strengths of glass-infiltrated alumina ceramics were prepared using various surface treatments. Shear bond strength test was conducted to determine the effect of surface treatment methods on bonding among the zirconia or the veneering porcelain and the composite resin.

Previous studies proved that the mechanical conditioning that creates microretentions and improves adhesion has been efficient in surface treatment of zirconia and veneer ceramic material [31, 32]. In clinical condition, while the fractured restoration is repaired intraorally, the cracked surface is usually ground using a coarse diamond bur, which is a common procedure to be followed by dentists, in order to enhance mechanical bonding, because of cleaning remnants and smoothing the surface on the contaminated area [7, 33]. Grinding with diamond bur is selected as the control group. Al_2_O_3_ abrasion is another common treatment that is used to increase surface roughness, but there is no consensus on the bonding effectiveness to zirconia among researchers. Some of them stated that sandblasting caused superior bonding to zirconia, while rest of the others reported that this method did not significantly ascend the bonding strength between the zirconia ceramics and the composite.

Recently, investigators have focused on the use of laser treatment to roughen the surface of ceramic substrates. Despite many studies [17–20, 33–35] related to Nd-YAG, Er-YAG, and CO_2_ lasers in terms of surface conditioning of ceramics have been published, the data including the influence of Er,Cr:YSGG laser on the bond strength of composite resin to ceramic, zirconia, or 50% ceramic-50% zirconia surfaces are scarce. Thus, grinding with coarse diamond bur, sandblasting with Al_2_O_3_ particles, and laser treatment via Er,Cr:YSGG were preferred to be used in this study.

It is evident from the present study that grinding with diamond bur resulted in the highest shear bond strength values among other subgroups. The clinically acceptable level of shear bond strength between composite resin and ceramic materials has been reported in previous studies as 10-13 MPa. In the current study, the mean bond strength values were around the range of the recommended level, except those determined for short- and long-pulsed laser treatments applied on the zirconia surfaces. However, it should be taken into account that oral environment, mastication patterns, and long-term usage of the restoration were not able to simulate in the study, which can be caused to decrease on bond strength of the repair system over time. While the in vitro results cannot be exactly depicted to the in vivo conditions, the findings are beneficial predictor of a material's performance or a treatment method.

For Group I that consists only zirconia specimens, grinding with diamond bur showed higher mean shear bond strength value than other surface treatment methods, but only the differences between grinding and laser treatments were statistically significant. The possible reason for that finding is the rougher surface areas that can be confirmed by the SEM images for either grinding or sandblasting. While the surface roughness increase, it can result in not only higher surface area but also ascending surface energy. First of them enables micromechanical retention, and the latter can lead to wettability and adhesion.

According to our findings in this study, these were compatible with those presented in the study of Derand and Derand [36], in which highest bond strength value between luting cements and zirconia was obtained with diamond bur compared to no treatment, sandblasting, and hydrofluoric acid treatment. That study attributed the highest bond strength to more surface irregularities which were characterized by almost parallel ditches. The SEM examination in this study revealed that grinding caused surface roughness similar to sandblasting instead of creating pronounced parallel ditches. This can be due to usage of burs having different microstructures. As can be observed in the study of Kumbuloglu et al. [37], different burs (K1 and special Voco burs) created various surface patterns on the glass-infiltrated ceramic reinforced with lithium-disilicate.

Ghasemi et al. [23] presented that microshear bond strength between zirconia and resin cement improved when Er,Cr:YSGG laser output was set in 3 W. However, when the same type of laser with power outputs from 0.5 W to 5.0 W were used on the glass-infiltrate alumina blocks, no significant differences were reported among experimental groups by Eduardo et al. [21]. Furthermore, it was shown that 1.5 W and 4 W laser power output settings led to the highest microtensile bond strength. The SEM analysis of the zirconia specimens treated with Er,Cr:YSGG laser irradiation exhibited smoother zirconia surface compared to grinding and sandblasting treatments in the current study.

In the present study, lower values of shear bond strength were obtained applying short- or long-pulsed Er,Cr:YSGG laser treatments than sandblasting and control groups on zirconia surfaces. It can be considered that the power output setting of 1.5 W used in this study could be insufficient to modify and roughen the zirconia surface. It has been conferred that the lower energy settings created minor changes on the surface of Y-TZP, while the higher parameters showed up excessive deterioration on the material, with strong occurrence of cracks and carbonization [38].

The failure mode analysis demonstrated different fracture patterns for zirconia specimens. All specimens in laser-treated groups had adhesive fractures, whereas some specimens in grinding and sandblasting groups had mix failures. This can be predicted as another indicator of the insufficient performance of Er,Cr:YSGG laser conditioning on zirconia surfaces compared to other surface methods. It may be suggested that zirconia samples irradiated with Er,Cr:YSGG laser do not guarantee a convenient clinical service.

Irrespective of the laser pulse duration, the difference between grinding and laser treatments was statistically significant, whereas the difference between sandblasting and laser treatments was insignificant. There was inconsistency of the findings compared to the results of research paper of Ghasemi et al. [23], in which sandblasting demonstrated significantly higher microshear bond strength between the zirconia and resin cements than Er,Cr:YSGG laser application. The disagreement could be attributed to the differences in both the sandblasting techniques and the laser irradiation power settings, since only a 1.5 W power setting was used in the present study.

On the other hand, the study of Kirmali et al. [22] reported no statistically significant difference between Er,Cr:YSGG laser application and sandblasting in terms of repair bond strength zirconia, which was in accordance with the current study.

Higher shear bond strength values were achieved for specimens involving veneering ceramic surfaces, irrespective of the surface treatment methods used in this study. It could be attributed to higher vitreous proportion in ceramic than zirconia [39]. Since microretention has not been sufficient alone to achieve bonding that is a clinically acceptable, various adhesive systems have been put into market to create a well-organized chemical union to veneer ceramic and zirconia [30]. It has been shown that conventional silane coupling agents do not have potential for chemical bonding to the zirconia. A phosphate containing hydropically stable monomer, called MDP, improves the adhesion between composite resin and zirconia ceramic [14]. The adhesive system (Clearfil Repair) used in this study contains both silane and MDP monomer, to provide chemical bonding to veneer ceramic and zirconia, respectively. In this respect, Ghavam et al. [40] suggested that solely application of Vertise flow is suitable to adequate bonding onto zirconia surfaces.

In this study, the mean shear bond strength values obtained between the Clearfil Repair system and pretreated veneering ceramic surfaces in terms of grinding with bur and sandblasting were 16.76 ± 5.79 MPa and 12.01 ± 2.49 MPa, respectively, and no statistically significant difference was found. These results were in agreement with those reported in the study of Jain et al. [40], in which the Clearfil Repair system has the mean shear bond strength values of 14.03 ± 2.32 MPa and 14.64 ± 2.28 MPa for ceramic substrate in the sense of conditioning with grinding using a diamond bur and air abrasion, respectively, after thermocycling was performed. Additionally, Jain et al. [41] reported no statistically significant differences between sandblasting and grinding with bur related to the shear bond strength of the Clearfil Repair system to ceramic substrate, which is consistent with the findings of our study.

It seems that short- or long-pulsed Er,Cr:YSGG laser application is a more effective surface treatment procedure for ceramic surfaces than zirconia surfaces, because no statistically significant differences were determined between laser treatment and other methods in Group III which consists specimens comprising only ceramic surfaces. For Group II, comprising ceramic and zirconia surfaces, even though the mean bond strength value found for grinding with diamond bur was the highest, the difference between short-pulsed laser and grinding was not statistically significant.

## 5. Conclusions

Within the limitations of the current study, the following conclusions could be reported:

1. Surface conditioning using grinding with diamond bur provided better repair bond strength for each level of fractured zirconia ceramics
2. Sandblasting and Er,Cr:YSGG laser applications obtained appropriate bond strength for 50% veneering ceramic-50% zirconia surface, due to no significant differences observed among the applied surface conditioning methods in this group
3. The recommendations of manufacturer should be followed to acquire adequate bond strength between the composite resin and the cracked zirconia ceramics
4. The veneering ceramic included surfaces illustrated dominantly cohesive or mix failure modes. Intact veneering ceramic surfaces may be included into the repairing zone, in order to enhance bonding strength.

## Figures and Tables

**Table 1:** Mean and standard deviations of shear bond strength at the groups/subgroups.

Groups/subgroups	Group I (zirconia)	Group II (veneering ceramic)	Group III (zirconia/veneering ceramic)
Subgroup I (control-grinding)	12.94 ± 4.27^a^	16.76 ± 5.59^a^	16.6 ± 5.48^a^
Subgroup II (sandblasting)	11.35 ± 4.32^ab^	12.01 ± 2.49^b^	12.87 ± 1.67^a^
Subgroup III (long-pulse laser)	8.09 ± 1.53^b^	9.34 ± 3.16^b^	14.55 ± 2.99^a^
Subgroup IV (short-pulse laser)	7.61 ± 3.11^b^	12.46 ± 2.48^ab^	13.78 ± 2.51^a^

**Table 2:** Failure modes on cracked surfaces of tested groups/subgroups.

Groups/subgroups	Group I (zirconia)	Group II (veneering ceramic)	Group III (zirconia/veneering ceramic)
Subgroup I (control-grinding)	8 adhesive failure 2 mix failure	9 cohesive failure 1 mix failure	4 cohesive failure 1 adhesive failure 5 mix failure
Subgroup II (sandblasting)	8 adhesive failure 2 mix failure	8 cohesive failure 2 mix failure	4 cohesive failure 6 mix failure
Subgroup III (long-pulse laser)	10 adhesive failure	7 cohesive failure 1 adhesive failure 2 mix failure	4 cohesive failure 6 mix failure
Subgroup IV (short-pulse laser)	10 adhesive failure	7 cohesive failure 1 adhesive failure 2 mix failure	2 cohesive failure 8 mix failure

## Data Availability

The data used to support the findings of this study are included within the article.
